# Sexual dysfunction as a challenge in treated breast cancer: in-depth analysis and risk assessment to improve individual outcomes

**DOI:** 10.3389/fonc.2022.955057

**Published:** 2022-08-02

**Authors:** Abraham Hernández-Blanquisett, Valeria Quintero-Carreño, Angelina Álvarez-Londoño, María Cristina Martínez-Ávila, Raissa Diaz-Cáceres

**Affiliations:** ^1^ Cancer Institute, Centro Hospitalario Serena del Mar, Cartagena, Colombia; ^2^ Department of Psychology Oncology, Centro Hospitalario Serena del Mar, Cartagena, Colombia

**Keywords:** breast cancer, female sexual dysfunction, breast cancer survivor, risk factors, sexology, risk assessment, patient stratification

## Abstract

The increasing number of breast cancer survivors has led to a greater emphasis on issues related to quality of life (QoL). Up to 75% of women treated for breast cancer (BC) report sexual disorders. However, most oncologists are not trained to recognize which patients are at high-risk of developing sexual disorders. Female sexual dysfunction (FSD) is common in patients with BC; we found that patients without FSD prior to BC treatment are at risk of developing FSD after treatment. Treatment of early BC relies on the combination of chemotherapy, surgery, and radiation therapy. All these treatments have side effects or sequelae identified as high-risk factors for the development of FSD. The choice of less toxic treatments in each modality could reduce the risk of FSD in some cases, without affecting the risk of recurrence or effectiveness. A comprehensive approach of BC must consider FSD as a determinant factor of QoL in survivors.

## Introduction

Breast cancer (BC) is the most common cancer in women worldwide, with more than 2.2 million cases in 2020 (1), it is the leading cause of cancer mortality in women in the world. According to figures from the World Health Organization (WHO) in 2020, around 685,000 women died as a result of BC, with most deaths occurring in low and middle-income countries (1). Fortunately, with early diagnosis and proper treatment, BC patients can have 5-year survival rates of 80-90% (2). The increasing number of survivors has led to a greater emphasis on issues related to quality of life (QoL) in BC survivors.

The treatments of BC have particular consequences for the female body, which can directly influence a woman’s self-esteem, appearance and sexual desire (3, 4). Despite efforts to comprehensively address survivorship issues in these patients, several concerns remain unaddressed, particularly those related to sexual function (3, 4). Most oncologists are not trained to advise, carry out preventive interventions or treat patients with disorders related to alterations in sexual function, mainly because of the lack of education on this topic during oncology training. Reports indicated that many doctors are uncomfortable or ill-prepared to address female sexual dysfunction (FSD) (3). Up to 75% of people treated for BC report temporary or permanent physical, psychological, or interpersonal sexual concerns (4) and sexual disorders, as well as disorders related to anxiety, pain, fatigue, marital life, and their overall QoL (3, 5). However, in daily practice it is unusual for BC survivors to be referred to sexologist to assess these aspects at the end of treatment.

?>In short, addressing concerns about sexuality and intimacy are paramount issues in the care of BC survivors, who must be in the care of a specialist in sexologist for the comprehensive management of FSD (5).

In this manuscript, we review the literature on BC and sexuality to describe the risk factors most frequently related to FSD after breast cancer diagnosis and treatment. Based on this research, we developed a checklist that could be helpful in identifying patients at high-risk for FSD and could help oncologists refer high-risk patients accurately.

## Early BC current treatments

The fundamental pillars of local treatment for BC are surgery, chemotherapy, and radiotherapy. Over the course of several decades, the surgical treatment of BC has evolved significantly, from radical mastectomy to breast conserving surgery (lumpectomy), with the intention to minimize the cosmetic and functional sequelae (6). The main goal of breast conservation therapy is to provide primary tumor control and to preserve an acceptable cosmetic appearance of the breast comparable to mastectomy (6).

Today, the standard management of early-stage invasive BC and carcinoma *in situ* is lumpectomy with whole breast radiotherapy (WBRT), which is equivalent in terms of overall survival and local recurrence to radical mastectomy. In these patients, radiotherapy reduces the risk of local recurrence by 60-70% in invasive tumors and by 50-60% in tumors in situ (7–9). This is considered a remarkable achievement of modern oncology since previously, all women with BC, regardless of the stage of their disease, were treated with a radical mastectomy, which increases the incidence and severity of depression and anxiety in these patients (10). Additionally, studies have shown that women undergoing lumpectomy are more likely to sustain feelings for their physical attractiveness (11). The remaining percentage of breast tissue contributes to better breast-specific sensuality; being an important part of sexual desire during intercourse, intimacy and/or the experience of pleasurable breast caressing (11). This seems to correlate with improved sexual function postoperatively (11).

Regarding BC in localized advance-stage cancer, scientific evidence supports the use of neoadjuvant chemotherapy (NCT) mainly based on Anthracyclines and Taxanes, to reduce the size of the tumor so that the patient can be managed with conservative surgery and WBRT (12–14). If this is not achieved, the indicated treatment will be mastectomy and chest wall radiation, since the latter provides a benefit in terms of local control with a decrease in the relative risk of recurrence of 60-70% and an improvement in terms of overall survival of 10% (12–14). Despite the indisputable clinical benefits of NCT, unfortunately, in most cases there are significant toxicities such as alopecia, neutropenia, nausea, vomiting, anemia and premature ovarian failure. In addition, some side effects such as chronic fatigue, neurotoxicity, cardiotoxicity or FSD can appear months after treatment. In this context, Anthracycline-free regimens are an interesting option to consider, since they have shown less toxicity with equal efficacy (15).

On the other hand, the most commonly injured organs during radiation therapy treatment in BC patients are skin, heart, and lung; and, if regional nodal irradiation is added, secondary effects may occur in the brachial plexus and shoulder (6). At present, with the development of modern techniques, radiotherapy generally does not generate significant acute and late toxicity that impairs QoL. However, the cosmetic changes that this can generate on the irradiated breast continue to be a great concern due to the subsequent emotional impact that patients can experience and report. Worse cosmetic results have been described with treatment decisions as doses greater than 50 Gy, addition of boost, regional nodal irradiation, inhomogeneity of the radiation dose, more than two fields and no use of compensation filters (6). Likewise, other characteristics such as breast size, age, race, extent of surgical resection, scar orientation and chemotherapy can also affect aesthetic results and self-perception of the body, and therefore must be considered in treatment selection (6).

## Sexual education in the medical school

For medicine faculties, sexual education focuses on birth control, anatomy, and physiology of the reproductive organs (16). Studies have shown that sexual health education among undergraduate medical students predominantly focuses on reproduction and organic diseases (70%) (17). Likewise, a survey carried out with 125 medical students reveals that 53.8% were afraid of offending the patient asking about sexual health topics and 33.4% admitted insufficient knowledge on the topic (17).

Sexual supportive care should be discussed and taught as an integral part of training in medical school concerning sexual education and rights in all patients, especially in oncological patients. Sexual education must be taught during medical school and up to all the specialists dealing with sexual health problems in clinical practice (urology, obstetrics and gynecology, psychiatry, endocrinology, oncologist) (16, 18). However, this does not happen in most medical schools and training programs in most countries around the world. In Latin America for example, a survey demonstrated no more than 18% medical schools provide some type of modern instruction on sexual education (19). Among 366 participants from 40 countries in Europe, 62.3% surveyed had not received any training in sexual health and 48,1% did not have it as a part of their curriculum (18). Literature corroborates the lack of sexual education as a tangible reality.

## Sexual functioning and BC patients

The WHO defines human sexuality as a permanent and continuous variable throughout the life cycle (20). It includes sex, gender identity, sexual orientations, eroticism, subjective sexual arousal, pleasure, intercourse, intimacy, and reproduction as main categories, which are reinforced through social, historical, cultural, psychological, ethical, legal, historical, spiritual, religious construction, experienced through the belief system of each individual immersed in a culture or social group (20, 21).

From the traditional physiological model, Master and Johnson represented sexual response as a linear state that includes the phases of arousal, plateau, orgasm, and resolution (22). In men, there is also a refractory period (22). Later, Kaplan (1979), recognized a new phase in this interpretation of sexual functioning by assigning the variable desire or sexual interest defined as an emotion, an impulse that causes motivation to initiate and continue establishing interpersonal relationships accompanied by the sensation of intimacy and affection for the full enjoyment of pleasure through the human potentiality of eroticism (23). Sexual intimacy and consummating erotic desire are then phases associated with the perception of affective responses as a fundamental part of sexual satisfaction, the latter being the result or state of gratification reinforced through sexual communication between the couple (20, 24).

Another model of sexual response that strays from a purely biological explanation, which includes desire, is Basson’s cycle of sexual response in women (25). According to Basson’s model, female sexual function is found in the context of awareness of non-sexual needs that are explained through the bond of emotional intimacy, affection, commitment, tolerance, and perhaps sexual activity (25). Thus, sexual functioning is interpreted through a deliberate decision to choose a sexual stimulus that could lead to physical experience, subjective arousal, and the possibility or not of having an orgasm (25).

As has been observed so far, sexual functioning requires several variables that lead to the recognition of a free enjoyment of sexuality, requiring a synchrony between organic, psychological, affective, behavioral, cultural, and other dimensions. The diagnosis of BC, its symptomatology, and the respective treatments, influence the patient’s perception of their QoL (25). Concern about the possibility of being mutilated, the risk of dying, the prolonged presence of pain and the distortion of their body image are risk factors that trigger anxious and depressive symptoms, suffering or distress (25).

In this aspect, the diagnosis, and some type of treatments for BC will be risk factors for sexual health impairment and FSD, in addition to physical or mental health alterations. The Netherlands Cancer Institute in Amsterdam conducted a study with 169 BC survivors and 67 partners to identify the correlation between sexual dysfunctions and BC in women, showing a prevalence of hypoactive sexual desire disorder in 83% of cases, followed by sexual arousal disorder with 40% and finally a 33% incidence for a diagnosis of dyspareunia (26). Dyspareunia affects female sexual health by alterations in sexual satisfaction, orgasmic functioning, and the appearance of pain during sexual intercourse (26). The perception of declining QoL is interpreted with the presence of sexual dysfunctions. For this reason, sexuality should be considered fundamental in therapeutic decisions following a diagnosis of BC. The question is timing: when should we refer to the specialist? Most patients do not complain of sexual health problems at the time of diagnosis (27, 28), conversely the simultaneous monitoring of a multidisciplinary care group that includes sex therapists, sexologists or clinical sexologists could prevent the risk of sexual dysfunction (29, 30).

The recommendation for the integral management of female sexuality and BC seeks to promote an intervention for the reinterpretation of the sexual response based on sexual desire and satisfaction, its reorganization before the possibility of sexual activity without penetration, the use of lubricants and vibrators as non-pharmacological strategies, providing counseling and couples therapy as protective factors to resume intimacy and sexual communication (27).

## Identifying which patients are in high-risk of sexual dysfunction

BC patients face great challenges in their lives. Once their BC doctor utters those terrible words, their world is turned upside down. As clinicians, our goal is to fight the disease in all its aspects; modern oncology goes beyond the simple prescription of chemotherapeutic agents. The multidisciplinary approach is the gold standard for the treatment of BC, which involves specialists from multiple areas in the diagnosis, treatment, rehabilitation, and follow-up. Nevertheless, there are some holdovers from the old days where BC doctors remain oblivious to some situations that are vital to patients. Sometimes sexuality is the last topic to be addressed in follow-ups, but FSD directly impacts a patient’s mood and subsequent recovery from cancer (17, 28). As mentioned above, many BC specialists are not adequately trained to recognize and treat FSD associated with BC treatments. In this context, we reviewed the literature to find what factors could identify those patients at high-risk for sexual dysfunction and we put them together into one simple checklist that could help clinicians address the problem easily and in a timely manner.

Below, we present the main risk factors related to sexual dysfunction in BC patients in a checklist format (**Table 1**). Other widely known risk factors for sexual dysfunctions, such as chronic severe diseases, endocrine disorders, obesity, smoking, hypertension, psychiatric disorders, hysterectomy, age, marital status, sexual orientation, or sociocultural factors were grouped under the category of others risk factor for FSD not related to BC (29, 30). Because these factors are not directly related with BC treatment.

**Table 1:** Risk factors for female sexual dysfunction in breast cancer survivors.

	YES	NO
**Mastectomy**		
**Chemotherapy**		
**Aromatase inhibitors**		
**Use of concomitant medication (SSRis/SNRis)**		
**Chronic pain**		
**Other risk factors for FSD not related to breast cancer**		

## Selected Risk factors

### Mastectomy

In 2014, L Aerts et al. conducted a prospective controlled study comparing the impact of mastectomy versus breast-conserving surgery on the sexual functioning of 149 BC patients compared to 149 age-matched controls. The median age was 57 years and most of the patients were cohabiting or married patients. In this study, 68 patients were treated with mastectomy and 81 with conservative surgery. In the month prior to surgery, 76% of the breast-conserving surgery group and 50% of the mastectomy group were sexually active. The Dyadic Adjustment Scale (DAS) was used to assess the quality of the couple’s relationship, to measure the impact on sexual functioning, two questionnaires developed by the authors were used. The results showed no difference between healthy patients and those who underwent conservative surgery for BC. However, compared to healthy women, women in the mastectomy group show more problems in sexual desire and sexual arousal six months after surgery and more problems in sexual desire, the ability to achieve orgasm, and a lower intensity of orgasm 1-year after surgery. These differences were statistically significant (31).

### Chemotherapy

Premature ovarian failure caused by chemotherapy results in decreased estrogen levels and is a known cause of vaginal dryness and dyspareunia. In 2002, Ganz et al. showed the results of a retrospective follow-up of 817 BC survivors, where sex life was significantly worse in women who received chemotherapy compared to women who received tamoxifen (32). Another study in 2017 reported that anti-Müllerian hormone levels are undetectable in most women receiving chemotherapy and, more importantly, remain at low levels after completion of treatment in most women (33). In addition, in 2021, Qi et al. published a retrospective study of 201 women <50 years without FSD prior to treatment, who were evaluated after finishing their treatment for BC. Unfortunately, 83% documented the appearance of sexual dysfunction. In the multivariate analysis, chemotherapy was found as an independent risk factor for FSD (OR 11.876). In addition to total mastectomy (OR 7.84) and endocrine therapy (OR 19.688) (34).

### Aromatase inhibitors

In 2011, Panjari et al, conducted a retrospective study of 1,684 BC patients enrolled in the BUPA trial and assessed their sexual function using the Menopause-Specific Quality of Life Questionnaire, a set of 5 questions “yes/no” scores on libido were included to determine whether low libido was prior or secondary to breast cancer and treatment. Patients older than 70 years, with active disease, widowers and without a partner were excluded from the final analysis. The authors found that prior to diagnosis, 82.7% of the patients had good and satisfactory sexual function. At the time of the questionnaire, 70% of the patients reported sexual function problems. In this study, women taking aromatase inhibitors were 1.5 times more likely to report sexual function problems (OR 1.50, 95% CI 1.0, 2.2, P = 0.04), while women using tamoxifen did not (OR 1.1, 95% CI 0.8, 1.5, P = 0.6) (35).

### Use of concomitant medication

Selective serotonin reuptake inhibitors (SSRIs) and serotonin and norepinephrine reuptake inhibitors (SNRIs) used for hot flashes secondary to tamoxifen or anti-estrogen therapies can cause reduced libido, altered excitement and anorgasmia. Reducing the dose or changing to a different drug could be helpful in these cases, because the effect may be dependent on the dose of the drug (36).

### Chronic pain

With advances and greater effectiveness in the treatment of BC, there are more survivors of this disease, and because of this, many women are left with a lasting legacy of chronic pain, which has a significant impact on their functionality, physical health, sexual, emotional and in general in their QoL. Chronic pain, generally of the neuropathic type, is reported in the literature as the most frequent complication in BC survivors (37, 38). Chronic pain after BC surgery has traditionally been called Postmastectomy Pain Syndrome (PMPS) (39). However, this term can be misleading, since persistent pain can also develop after breast-conserving surgeries, therefore nomenclatures such as postoperative breast pain or persistent pain after BC surgery are also used (39). The International Association for the Study of Pain defines PMPS as chronic pain (greater than three months of evolution), non-malignant and that does not stop immediately after BC surgery, affecting the anterior chest, armpit and/or the upper medial aspect of the arm (37, 40). Incidence rates for persistent pain after BC surgery vary in the literature, with reports ranging in their estimates from 11-57% (39).

Another type of painful condition described after breast surgery is phantom breast syndrome, which comprises a set of symptoms that occur in the absent breast (37, 41). These symptoms range from intensely painful phenomena to simple discomfort or non-painful sensations such as itching, throbbing, pressure, or a tingling sensation, which occur in 30-80% of women after mastectomy (41). Anxiety or stress can worsen this clinical picture. Therefore, it is very important that the doctor inquiries about these symptoms in order to offer therapeutic options to patients that allow them to improve their QoL.

The therapeutic approach to these types of pain described should be based on multimodal pain treatment, generally, it is carried out according to the analgesic ladder of the WHO (42), associating adjuvant treatment, neuromodulators, physical therapy and in some cases interventional management.

## Discussion

Education and sexual health should be considered a mainstay in the care of cancer patients in general. It is necessary to ask, instruct and encourage sexual practices and provide safe environments to freely discuss this issue during the consultation. Just as we take the time to explain to our patients how and when to take their oral chemotherapy pills, we must also prescribe, counsel, and encourage safe sexual practices. We must always remember that sexuality is an important issue for the health and QoL of women.

FSD is common in patients with BC; in our review we found robust evidence that patients without FSD prior to BC treatment are at risk of developing FSD after treatment. Treatment of early BC currently relies on the combination of chemotherapy, surgery, and radiation therapy. All these treatments have side effects or sequelae that have been identified as high-risk factors for the development of FSD. Nonetheless, when deciding the ideal treatment for each patient, the risk of FSD is not normally considered, nor is it specifically recommended in international clinical practice guidelines.

Treatment course sometimes use the combination of 3 or more options that are considered of high-risk for the development of FSD. For example, NCT regimens, followed by mastectomy, radiotherapy, hormonal therapy with aromatase inhibitors is common. What, if it is worth discussing, the choice of less toxic treatments in each modality? For instance, consider the use of short and Anthracycline-free chemotherapy regimens, chosing conservative surgery, when possible, prescribe less toxic radiotherapy techniques such as IMRT and avoiding the risk factors associated with worse cosmetic result, described in the treatment section.

We believe that a better selection of treatment options could, in some cases, reduce the risk of FSD. In addition, early referral to an expert in sexology could reduce the impact on QoL and sexual life of those patients who already have high-risk factors at the initial visit or post-treatment visit. However, in our daily practice it is rare for patients with high-risk factors to be referred to an expert in sexology, unless they developed FSD. We believe that this is mainly due to two factors:

1) The lack of proper training for doctors in matters of sexology that leads to the non-recognition of sexual health as a vital important issue. Bearing this in mind, it would be important for the health-care institutions to include in their educational programs in medicine and areas related to oncology more training on issues of sexology.

2) The non-perception of the BC treatments carried out as high-risk factors for FSD. This point was what motivated us to design a simple checklist where doctors can quickly consult if the patient has a high-risk of FSD and with this select a better treatment strategy (if possible) in addition to referring the BC survivor to a specialist in sexology at the right time.

## Conclusions

The evaluation of FSD is of great relevance. The identification of specific needs for the cancer patient will improve the QoL in that difficult stage. Education and sexual health should be considered a pillar in the care of patients with cancer. The right moment to approach sexuality is a great challenge in daily practice and a good relationship with the patient is essential, knowing risk factors could help oncologists refer high-risk patients on a timely basis. We need to learn to approach both cancer and sexuality with compassion.

## Author contributions

MCMÁ and RDC reviewed the literature and drafted the article. VQC and AAL conceived the review and drew the tables. AHB revised the manuscript. All authors read and approved the final manuscript.

## Funding

Author’s resources.

## Conflict of interest

The authors declare that the research was conducted in the absence of any commercial or financial relationships that could be construed as a potential conflict of interest.

## Publisher’s note

All claims expressed in this article are solely those of the authors and do not necessarily represent those of their affiliated organizations, or those of the publisher, the editors and the reviewers. Any product that may be evaluated in this article, or claim that may be made by its manufacturer, is not guaranteed or endorsed by the publisher.
